# History of co-occurring disorders and current mental health status among homeless veterans

**DOI:** 10.1186/s12889-018-5700-6

**Published:** 2018-06-18

**Authors:** Kele Ding, Matthew Slate, Jingzhen Yang

**Affiliations:** Kent State University, Kent, OH USA

**Keywords:** Homeless veterans, Co-occurring disorder, Mental health, Community health

## Abstract

**Background:**

Homeless veterans are at high risk for co-occurring disorders (COD), defined as mental illnesses that include at least one alcohol or other drug use disorder and at least one non-drug related mental disorder. However, epidemiological studies examining the prevalence of COD and associated mental health status in this population are limited. The objectives of the study were: (1) to describe a history of diagnosed mental disorders among homeless veterans admitted to a transitional housing program, and (2) to examine the associations of the prior diagnosed COD and other mental disorders with current mental health status.

**Methods:**

Study participants were male homeless veterans admitted to a transitional housing program from July 2015 to September 2017 in a large municipal area in Northeast Ohio, the United States. Cross-sectional, self-reported data from the admission assessment were included and analyzed. History of mental disorder diagnoses were aggregated into five categories for the purpose of this study: no mental disorders, only alcohol or other drug use disorder(s), one non-drug related mental disorder, two or more non-drug related mental disorders, and COD. Current mental status were measured as empowerment, mental component summary score (MCS) and physical component summary score (PCS) of health related quality of life (VR-12), and perceived overall well-being. Sample distribution of the five categories and their associations with current mental status were examined using Generalized Linear Model test.

**Results:**

Of all participants, 76.7% had at least one prior diagnosed mental disorder, including 47.4% with any drug-related disorders. Over one-third (37.2%) reported having COD. Compared to those with no mental disorder history, those with COD scored significantly lower on MCS and empowerment scores; those with any prior diagnosed non-drug related mental disorders also scored significantly lower on MCS. No significant differences, however, were found in current mental health status between those with COD and those with mental disorders but not COD.

**Conclusions:**

COD prevalence among homeless veterans was within the parameter of other literature reports. Veterans with COD compared to veterans with no history of mental disorders tended to have lower MCS and empowerment scores. Veterans with COD had the same mental health status as those with other mental disorders.

## Background

Co-occurring disorders (COD) refer to mental illnesses that include at least one alcohol or other drug use disorder and at least one non-drug related mental disorder that occurs simultaneously or in a different timeframe to the same person [1–3]. Compared to a single diagnosed mental disorder (including alcohol or other drug abuse disorder), individuals with COD require more treatment attention, are prone to complications, and are at greater risk of experiencing negative consequences, including posttraumatic stress disorder, psychiatric symptoms, poor personal hygiene, medication non-adherence, and/or violent behaviors [1, 4–6].

Homeless veterans are high risk for experiencing mental disorders. The prevalence of mental disorders among homeless veterans vary from 48 to 67%, according to a report from the Department of Veteran Affairs (VA) [7]. These percentages were double the percentages of domiciled veterans (21–34%) [8]. Previous studies also show that, in the homeless population, the prevalence of substance abuse ranges from 41 to 84% [9], and the prevalence of COD ranges from 20 to 50% [8, 10–12]. However, data on the prevalence of COD among homeless veterans are lacking. A survey study that examined the mental health conditions among male homeless veterans found that the percentages with two or more mental health conditions was 33.1%, and the percentages with alcohol or drug abuse/dependence was 79.5% [4]. However, the authors did not specify the percentage of COD among the homeless veterans.

In recent years, COD has been being increasingly recognized as a contributing factor to poor physical and mental health [13, 14]. However, most studies on COD among the homeless population have focused on treatment interventions or case management [15–22], or health outcomes that are not specific to veterans [21–25]. Studies examining the prevalence of COD and associated health status among homeless veterans are urgently needed to inform community organizations, so that effective services can be developed accordingly.

The aims of the current study were: (1) to describe a history of diagnosed mental disorders among homeless veterans who were admitted to a community transitional housing program, and (2) to examine the associations of the prior diagnosed COD and other mental disorders with homeless veterans’ current mental health status. We hypothesized that a history of at least one diagnosed mental disorder (addiction or psychiatric/psychological disorder) would be positively correlated with poor mental health status, and that homeless veterans with a prior diagnosed COD would have worse mental health status than those without a history of mental disorders and those with mental disorders but not a COD.

## Methods

### Participants

Study participants were male homeless veterans admitted to a community veteran transitional housing program from July 2015 to September 2017 in a large municipal area in Northeast Ohio, the United States. The goal of the program is to provide temporary living, case management services, and a therapeutic intervention program. Admission to the transitional housing program was based on the veteran’s eligibility for the U.S. Department of Veterans Affairs’ Grant & Per Diem services [26], and income level that fell under the 35th percentile of the median for the Ohio county. Eligible study participants were all male homeless veterans admitted to the transitional housing program who received the intervention that integrated Expressive Art Therapy [27] for mental health recovery. This study was approved by the Institutional Review Board (IRB) of the lead author’s institution (IRB number: 17–378).

### Procedure

Male veterans admitted to the local transitional housing program were automatically enrolled in the Expressive Art Therapy intervention, and invited to participate in this study. After written consent, participants completed the self-administered entrance assessment conducted in person by a trained staff member within the first two days of admission. Following the entrance assessment, participants received a therapeutic workshop for one and a half hours per day, five days per week, for 12 weeks. At the end of the 12-week workshop, participants were asked to complete the second assessments. For the purpose of this study, only the cross-sectional data collected during the entrance assessment were analyzed. A total of 265 participants who completed the entrance assessment were included in this study.

### Variables and measures

***History of mental disorders*** were derived from responses to the question asking, “Has a professional ever diagnosed you with any of the following conditions?” Participants were asked to check all responses that applied, including anxiety disorder, bipolar disorder, insomnia or other sleep disorder, post-traumatic stress disorder (PTSD), substance abuse or addiction (alcohol), substance abuse or addiction (other drugs), depression, and other mental disorders. In this study, such drug or alcohol addiction/dependency and/or psychiatric/psychological disorders are broadly called mental disorders. For the purpose of this study, responses were aggregated into the following five mutually exclusive categories, referred to as mental disorder categories for this study: 1) *COD,* defined as prior diagnosis of at least one alcohol or other drug use disorder and at least one non-drug related mental disorder; 2) *One non-drug related mental disorder,* defined as prior diagnosis of one non-drug related mental disorder with no alcohol or other drug use disorder; 3) *Two or more non-drug related mental disorders,* defined as prior diagnosis of two or more non-drug related mental disorders with no alcohol or other drug use disorder; 4) *Alcohol or other drug use disorder,* defined as prior diagnosis of an alcohol and/or other drug use disorder with no non-drug related mental disorder; and 5) *No mental disorder,* defined as no prior diagnosis of alcohol or other drug use disorder nor non-drug related mental disorder.

***Current Mental Health Status*** in this study included perceived overall well-being, physical and mental components of health related quality of life, and empowerment. They are described as follows.

***Perceived overall well-being*** was assessed using The Arizona Integrative Outcomes Scale (AIOS) [28]. The AIOS is a one item, 100-mm visual analogue scale measuring global well-being. Participants were instructed to “Mark the line below with an X at the point that summarizes your overall sense of well-being for the past month.” with the low anchor being, “Worst you have ever been” and the high anchor being, “Best you have ever been.” For the AIOS, the mark was manually measured from the left, and to the nearest millimeter, to obtain a numerical value for the question, which was then divided by the length of the actual 100 mm line and multiplied by 100 to obtain a percentage for the analysis. The higher the percentage, the better the overall well-being.

***Health related quality of life*** was measured using the Veterans’ RAND 12 Item Health Survey (VR-12) [29]. The VR-12 is a short version of the Veterans RAND 36 Item Health Survey that was developed and modified from the original RAND version of the 36-item Health Survey version 1.0 (also known as the “MOS SF-36”). The VR-12 measures both physical and mental health domains, including general health perceptions, physical functioning, role limitations due to physical and emotional problems, bodily pain, energy-fatigue, social functioning, and mental health. Scores for each domain were calculated using the method provided by the VR-12 team, resulting in a Physical Component Summary Score (PCS) and a Mental Component Summary Score (MCS), with high scores suggesting better quality of life.

***Empowerment*** was measured by the Consumer Empowerment Scale [30], a 28-item scale that measures five domains: Self-esteem & self-efficacy, Power-powerlessness, Community activism and autonomy, Optimism and control over the future, and Righteous anger, on a four-point forced choice scale: strongly agree (4), agree (3), disagree (2), and strongly disagree (1). The mean score of the 28 items was used in model testing, with a higher score indicating a higher level of consumer empowerment.

***Demographic information*** was collected based on the Program Intake Form [31] which included date of birth, race, length of military service, service in a war zone, and marital status. Due to limited sample size, only age, race, and service in a war zone were included as variables in the analysis.

### Data analysis

Data were examined for missing answers, error codes, and incompletion when defining useable cases. Twelve participants who had missing values for two-thirds or more questions were excluded from the analysis. Thus, a total of 253 participants were retained for the analysis.

Descriptive analysis was conducted to describe demographics, and distributions of each of the five mental disorder categories. Average scores for each of the dependent variables (perceived overall well-being, PCS, MCS, and empowerment) were compared across the five mental disorder categories using Generalized Linear Model (GLM), adjusting for age, race, and service in war zone. The dependent variables were examined for normal distribution and outliers prior to the model test. Tukey post hoc tests were performed for pairwise comparisons. Two sets of GLM tests were conducted. One was to compare veterans with mental disorders to those with no mental disorders. Another as to compare veterans with COD to those with non-drug related mental disorders. Statistical significance was set at the *p* < 0.05 level. Analyses were completed using SAS (v9.3).

## Results

### Demographic characteristics of participants

Among the all-male participating homeless veterans (*n* = 253), about 20% did not report date of birth. Of the remaining cases (*n* = 199), 37.2% were younger than 50 years of age, 51.0% were aged 50 to 64, and 12.0% were aged 65 or older. The age range was 22 to 78. Missing cases in age were replaced by the average of known ages in the model test. The most commonly reported race was White (*n* = 149, 58.9%), followed by Black (*n* = 86, 34.0%). Nearly half (44.3%) of participants had served in the military for more than three years, nearly one-third (32.8%) served one to three years, and slightly over 10% (10.7%) served less than one year. More than two-thirds (*n* = 180, 71.1%) had served in a war zone. Of the total sample, 32.8% reported that they never married, 44.7% were divorced, and 19.4% were married.

### History of mental disorders

**Fig. 1:**
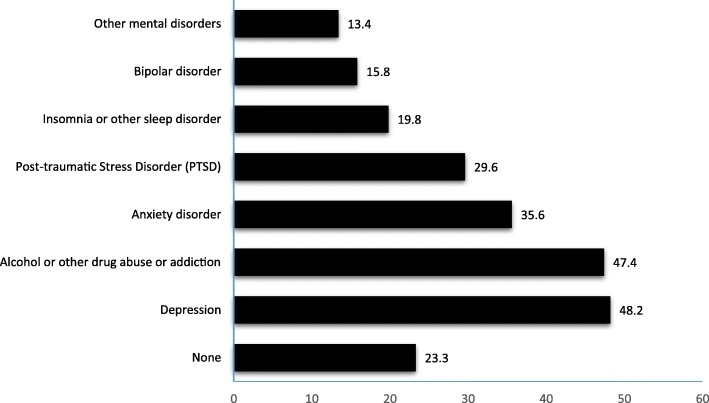
Distributions of Diagnosed Mental Disorders among participating homeless veterans (*n* = 253)

Of the 253 participating homeless veterans included in this analysis, 194 (76.7%) had at least one prior diagnosed mental disorder, including drug related addiction or dependency, and other psychiatric/psychological disorder, and 23.3% (*n* = 59) had no previously diagnosed mental disorders. Depression was the most commonly reported mental disorder (48.2%), followed by drug or alcohol abuse or addiction (47.4%). Figure 1 presents reported diagnosed mental disorders.

Of the 253 participants, 94 (37.2%) had COD (Table 1). Nearly one-fifth (*n* = 47, 18.6%) had two or more non-drug related mental disorders, and one fourth (*n* = 27, 10.7%) had one prior diagnosed non-drug related mental disorder. Table 1 presents the frequency distribution of reported mental disorders by COD category and two non-drug related mental disorder categories. The percentages in Table 1 are the percentages of a mental disorders in the mental disorder category. While 26 (21.7%) of those who had a diagnosed alcohol or other drug use disorder had not had any other mental disorders, this information is omitted from Table 1.

**Table 1:** Number and percent of diagnosed mental disorders by Mental Disorder Category^a^

Diagnosed Mental Disorders	Mental Disorder Category		
	One non-drug related mental disorder (*n* = 27)	Two or more non-drug related mental disorders (*n* = 47)	Co-occurring Disorders (*n* = 94)
Any substance abuse or addiction			94 (78.3)
Anxiety disorder	1 (1.1)	30 (33.3)	59 (65.6)
Traumatic brain injury (TBI)	0 (0.0)	6 (35.3)	11 (64.7)
Depression	11 (9.0)	35 (28.7)	76 (62.3)
Insomnia or other sleep disorder	5 (10.0)	15 (30.0)	30 (60.0)
Bipolar disorder	2 (5.0)	14 (35.0)	24 (60.0)
Other mental disorders	2 (5.9)	12 (35.3)	20 (58.8)
Post-traumatic stress disorder (PTSD)	6 (8.0)	25 (33.3)	44 (58.7)

^a^All percentages were row percentages in each own mental disorder type. Of the five mental disorder categories used by this study, No mental disorder (*n* = 59), and only Alcohol or other drug use disorder (*n* = 26) are omitted from this table display

### Current mental health status and the history of mental disorders

The mean scores for each of the dependent variables (overall well-being, PCS, MCS, and empowerment) are presented in Table 2, broken down by the five mental disorder categories. Descriptively, the mean scores were generally higher for participants with no mental disorders than those with a history of mental disorders, with the exception of the empowerment score. The model test suggests that, compared to participants with no mental disorders, participants with COD scored significantly lower on MCS and empowerment scores (*p* < 0.05), but had no significant differences in perceived overall well-being and PCS. Participants with one non-drug related mental disorder and participants with two or more non-drug related mental disorders had significantly lower scores on MCS compared to those with no mental disorder history (*p* < 0.05), but had no significant differences in PCS, empowerment, or perceived overall well-being.

**Table 2:** Mean score differences in mental health status by Five Mental Disorder categories (n = 253)

	No Mental Disorder	One non-drug related mental disorder	Two or more non-drug related mental disorders	Alcohol or Other Drug Use Disorder	Co-occurring Disorders
	(n = 59)	(n = 27)	(n = 47)	(n = 26)	(n = 94)
Perceived overall well-being					
Mean (95%CI^a^)	49.9 (40.36, 59.45)	41.8 (28.73, 54.84)	40.2 (29.94, 50.54)	47.4 (33.87, 60.89)	46.0 (37.65, 54.30)
*p*-value^b^	Ref	0.27	0.11	0.73	0.44
Physical component summary score (PCS)					
Mean (95%CI^a^)	44.1 (40.53, 47.67)	40.9 (35.84, 45.87)	41.1 (37.29, 44.88)	43.3 (38.28, 48.33)	41.6 (38.54, 44.72)
p-value^b^	Ref	0.25	0.21	0.78	0.22
Mental component summary score (MCS)					
Mean (95%CI^a^)	47.4 (43.62, 51.23)	40.5 (35.25, 45.85)	37.4 (33.35, 41.38)	44.6 (39.27, 49.90)	39.9 (36.65, 43.17)
p-value^b^	Ref	0.02	0.01	0.35	0.01
Empowerment					
Mean (95%CI^a^)	3.0 (2.89, 3.07)	2.9 (2.73, 2.99)	2.9 (2.79, 2.98)	3.0 (2.85, 3.11)	2.9 (2.78, 2.93)
p-value^b^	Ref	0.09	0.12	0.96	0.01

^a^95%CI = 95% confidence intervals. ^b^p values were based on generalized linear models adjusting for age, race, & service in war zone; multiple comparisons were made with No mental disorder as a reference group

When comparing the mental health status between participants with COD and with non-drug related mental disorders, no significant differences were found in the mean scores of any of the four mental health status variables (*p* > 0.05, Table 3).

**Table 3:** Mean score differences in mental health status between COD and non-drug related mental disorder categories

	Co-occurring Disorders (n = 94)	One non-drug related mental disorder (n = 27)	Two or more non-drug related mental disorders (n = 47)
Perceived overall well-being			
Mean (95%CI^a^)	46.0 (37.6, 54.3)	41.8 (28.7, 54.8)	40.2 (29.9, 50.5)
p-value^b^	Ref	0.55	0.31
Physical component summary score (PCS)			
Mean (95%CI)	41.6 (38.5, 44.7)	40.9 (33.8, 45.9)	41.1 (37.3, 44.9)
p-value	Ref	0.77	0.81
Mental component summary score (MCS)			
Mean (95%CI)	39.9 (36.7, 43.2)	40.5 (35.3, 45.9)	37.4 (33.3, 41.4)
p-value	Ref	0.82	0.28
Empowerment			
Mean (95%CI)	2.9 (2.8, 2.9)	2.9 (2.7, 3.0)	2.9 (2.8, 3.0)
p-value	Ref	0.92	0.52

^a^95%CI = 95% confidence intervals. ^b^p values were based on generalized linear model test adjusting for age, race, & service in war zone; multiple comparisons were made with Co-occurring disorders as a reference group

## Discussion

This study described a history of diagnosed COD and other mental disorders among homeless veterans participating in a local community transitional housing program, and examined the associations of prior diagnosed COD and mental disorders with current mental health status in the study population. The main findings show that 3 out of 4 (76.7%) homeless veterans have had at least one mental disorder (drug or alcohol addiction/dependency and/or psychiatric/psychological disorders). Over one-third of participants (37.2%) have experienced COD. Compared to homeless veterans with no mental disorder history, homeless veterans with a history of COD or other mental disorder(s) were at increased risk of poor mental health, especially on MCS and empowerment. No significant differences, however, were found in mental health status between those with COD and those with non-drug related mental disorders. Our findings provide a profile of a history of COD and other mental disorders among homeless veterans admitted to local transitional housing programs.

In a comprehensive analysis of VA administrative data, Rachel et al. [32] found that 21.0% of their participating veterans had mental illness (including substance abuse and dependency) [32]. Our observed prevalence of mental disorders was 76.7%, which is higher than the VA reports for homeless veterans (48–67%) [7]. The differences could be due to population variation; the current study was comprised of male homeless veterans only, had different race and marital status make up, and also restricted housing program inclusion criteria. The most prevalent prior mental disorders including COD in our study population may represent the more severe end of the mental disorder continuum, and thus, could have significant clinical implications for local programs that serve homeless veterans. The goals of these services are to provide opportunities for independent living, employment, and recovery from homelessness, including recovery from mental disorders.

The prevalence of prior COD diagnosis in this study population was 37.2%, which falls in line with the reported range of 20 to 50% in current literature [4, 8, 10–12]. It is worth noting that the reported mental disorders and COD in our study were based on self-reported prior diagnoses made by a professional. Even though the prevalence found here is within the reported range by literature, special attention to this population is needed. Veterans admitted to transitional housing programs do receive referrals for professional care about their drug use and dependency. However, they seek professional care on a voluntary basis. Currently, there is no clear mechanism that a transitional housing program could follow to monitor residents’ drug use and assistance in treatment. As the Substance Abuse and Mental Health Services Administration (SAMSHA) suggests, mental and substance use disorders are complex issues with biological, psychological, and social characteristics [33]. Integrated treatment and services should focus on multiple outcomes and include reduced substance use, improved psychiatric symptoms and functioning, decreased hospitalization, increased housing stability, fewer arrests, and improved quality of life. Considering that the length of stay in a transitional housing program could last up to one year or more for homeless veterans, an integrated drug rehabilitation service might provide additional support to recovery from homelessness.

Nearly half (47.4%) of participating homeless veterans reported history of substance abuse or addiction disorders, including alcohol and other drugs, which is in the low end of the range of 41 to 84% reported in the existing literature [4, 9]. Since most alcohol and other drug use rates in published studies were based on self-reported frequency and amount of substance use, they are different from the self-reported diagnosis of substance abuse or addiction disorders in this study. To ensure meaningful comparisons across studies, standardized measurement and data collection tools need to be used.

Our findings indicated that history of mental disorders was associated with a poorer mental health status. However, no significant differences in the mental health status were found between those with a history of co-occurring mental disorders and those with a history of one or more non-drug related mental disorders. This seemingly suggests that having a history of diagnosed mental disorder(s) could be associated with mental health status regardless of whether it is a COD or not. It is worthy to note that such a finding should not downplay the importance of COD as a mental disorder category of special attention. The impact of having a history of COD compared to having a history of other types of mental disorders included in this study, could be that it would complicate the treatment and intervention efforts for current mental health problems, and require special attention there, even though it makes no difference in current mental health status. This is of particular importance to local communities who serve homeless veterans.

### Limitations

There are several limitations worth noting. Our study population only included male homeless veterans admitted to a transitional housing program, and the sample size was small. Convenience samples and small sample size could threaten the internal and external validity. Thus, our findings may only reflect those admitted to our program. Reference to a general homeless veteran population should be made with caution. The cross-sectional survey nature of data collection increases potential for reporting and recall bias. Due to missing answers, the prevalence of prior diagnosis of mental disorders reported by this study might be underestimated because participants with missing answers are included in the denominator. The measures for mental health status were chosen due to the program goal and objectives, not specifically developed for a comparison study between COD and other mental disorders. There was a lack of detail about when the diagnosis was made, if formal treatment was provided, and if professional help was received by the time of the data collection. Nevertheless, mental disorders tend to be chronic.

## Conclusions

History of COD and other mental disorders are highly prevalent among the homeless veteran population. Even though literature has suggested the complexity of COD in treatment and care, in consequence or correlation with other behavioral and health indicators, this study did not find differences in the assessed mental health variables between homeless veterans with prior diagnosis of COD and homeless veterans with other types of mental disorders or mental disorder combinations. Our findings, however, do suggest that prior mental disorders are associated with poorer mental health status when compared to those without prior history of mental disorders. The impact that COD brings to our study population might be on the effectiveness of treatment and care, in that veterans with COD may need special attention when compared to those in other mental disorder categories.

## Abbreviations

AIOS: The Arizona Integrative Outcomes Scale
COD: Co-occurring mental health and a substance-use disorders
MCS: Mental Component Summary Score
PCS: Physical Component Summary Score
PTSD: Post-traumatic stress disorder
SAMSHA: Substance Abuse and Mental Health Services Administration
VA: Department of Veterans Affairs
